# Cochlear implantation in the world's largest medical device market: Utilization and awareness of cochlear implants in the United States

**DOI:** 10.1179/1467010013Z.00000000076

**Published:** 2013-03

**Authors:** Donna L. Sorkin

**Affiliations:** Executive Director, American Cochlear Implant Alliance, Virginia, USA

**Keywords:** Cochlear implant, Standard of care, Referral networks, Awareness, Utilization, Cost effectiveness

## Abstract

Provision of cochlear implants (CIs) for those within the criteria for implantation remains lower in the United States than in some other developed nations. When adults and children are grouped together, the rate of utilization/provision remains low at around 6%. For children, the provision rate is about 50% of those who could benefit from an implant, compared with figures of about 90% for the Flanders part of Belgium, the United Kingdom and other European countries. The probable reasons for this underprovision include: low awareness of the benefits of CIs among the population; low awareness among health-care professionals; the lack of specific referral pathways; some political issues relating to the Deaf Community; and financial issues related to health provision. Such financial issues result in situations which either fail to provide for access to implants or provide too low a level of the necessary funding, especially for low-income individuals covered by public health-care programs such as Medicaid. These issues might be mitigated by adoption and publication of standards for best clinical practices for CI provision, availability of current cost-effectiveness data, and the existence of an organization dedicated to cochlear implantation. Such an organization, the American Cochlear Implant Alliance (ACI Alliance), was recently organized and is described in the paper by Niparko *et al*. in this Supplement.

## Introduction

Since 1985, when the Food and Drug Administration (FDA) approved the first multichannel cochlear implant (CI) for use in the United States, the device has moved from being perceived as a somewhat controversial—even dubious—treatment for deafness to being recognized as a life-changing intervention for both adults and children. CIs are often characterized by recipients and their families as providing ‘miraculous’ outcomes. Research has demonstrated that cochlear implantation is highly cost effective (Cheng *et al*., 2000; Francis *et al*., 2002; Wyatt *et al*., 1996). Despite widespread acknowledgement of effectiveness and efficacy and as well as the fact that the intervention is covered by most public and private health insurance carriers in the United States, utilization by appropriate individuals remains low. It is estimated that fewer than 6% of Americans who could benefit from a CI have one (iData Research Inc., 2010).

This paper will: (1) explore the reasons for low utilization of CIs in the United States and (2) discuss what actions might help the CI intervention become more widely known and accepted as the appropriate treatment option for deaf individuals who meet FDA and Medicare candidacy criteria. Although most of the issues explored here are applicable to other countries, the specific information presented pertains to the United States.

## Standard of care

The term ‘standard of care’ is used in a number of disciplines including law, employment, business, and medicine. It refers to the watchfulness and caution that a reasonable person would utilize in a specific situation with regards to their duty and care. The parameters of a standard vary according to the circumstances. In a legal sense, the term refers to conduct that adheres to standards of behavior established by law for the protection of others against unreasonable risk of harm, or, in other words, negligence.

A person might be considered to be negligent if he or she acted in such a way as to have been the cause of harm to another individual. Under English law, this concept has evolved to include an implied promise to exercise care in the performance of services. For example, an innkeeper's duty in the performance of services includes the requirement to provide for the safety and security of guests.

In a contemporary framework, the standard of care in a business setting might reference what procedures a prudent company might follow in the manufacture of a specific product to avoid harm to others in the use of said product.

In a medical setting, standard of care refers to the formal diagnostic and treatment processes followed by a physician when a patient has certain symptoms or a specific disease or illness. A standard would encompass the guidelines and protocols considered most appropriate based upon scientific evidence. Sometimes such protocols are called ‘best practice.’ In general, if a particular medical treatment is considered best practice, we would expect that an individual would be evaluated for that treatment regardless of the type of health insurance they have or their ability to pay for the treatment.

Some common medical standard-of-care examples include testing and treatment options for diabetes; screening protocols for specific cancers such as breast, colon, and prostate; and heart disease screening procedures. All of these examples are well known among the medical and patient communities due to successful public awareness campaigns, consistent follow through by primary care physicians as part of routine care, and general adherence to suggested procedures among the general population.

Standards of care may evolve over a period of time and eventually become best practices. They may also be the result of clinical findings or even legislation. An example in which federal government legislation in the United States resulted in a standard of care being adopted is that of universal newborn hearing screening. The Newborn and Infant Hearing Screening and Intervention Act of 1999 was designed to establish a system of grants to encourage states to set up mechanisms to identify hearing loss in newborns and initiate programs for early intervention (EI). As a result of the federal legislation and the financial incentives that it provided, states moved quickly to establish mandatory screening programs. In 1997, prior to passage of the law, an estimated 7.5% of babies were screened by 1 month of age. Ten years later, the proportion of babies screened by 1 month of age had increased to 97% of all children born (CDC, 2010) moving newborn hearing screening from an ideal to a true standard of care. Newborn screening in the United States is now one of the tests performed routinely with babies during the first days of life.

A standard of care for medical treatment may vary by locality and sometimes between medical professionals within the same locality. Although standard of care is sometimes referenced in medical malpractice lawsuits, such references are not always made as it may not be appropriate to do so.

It is helpful to think about cochlear implantation within a standard-of-care context. Do diagnosis and typical treatment options for deafness include routine consideration of cochlear implantation? How familiar is the medical community and the general population with the intervention? How prevalent is the intervention among appropriate individuals? And given all of the above, is cochlear implantation the standard of care for severe to profound hearing loss in the United States? The discussion that follows addresses these questions.

## Severe to profound hearing loss and cochlear implantation

There were an estimated 34–36 million adults with measurable hearing loss in the United States in 2009 (Kochkin, 2009). Of that number, 1.2 million children and adults (with severe to profound hearing loss) were thought to be potential implant candidates (iData, 2010). The total number of CI recipients in the United States in 2009 was estimated at 70 000 adults and children (iData, 2010), yielding a utilization rate of 5.6% among the candidate population.

Given that utilization of hearing aids by people with hearing loss is only 20% (NIH, 2010), one might attribute the low CI utilization figure to similar behaviors (i.e. individuals not seeking appropriate hearing technology to address their hearing loss). In fact, hearing aid utilization is quite high among those with severe to profound hearing loss. It is estimated that 90% of people with profound hearing loss use amplification. For those with severe hearing loss, the utilization of amplification is 70%. Very low hearing aid utilization rates are typical of people with mild or moderate hearing losses, for whom utilization is estimated at 10 and 30%, respectively. Low hearing aid utilization rates are not typical of people with greater hearing difficulty (Gubler and Banziger, 2010).

## Demographics of the CI population

In most areas of the world, cochlear implantation is utilized to a greater extent by children than by adults. This is also true in the United States where the utilization rate for children with an appropriate audiological profile is approximately 50% compared to less than 5% utilization by appropriate adults. Reflecting a much larger base of adults (than children) with severe to profound hearing loss who are appropriate CI candidates, the mix of CI surgeries in the United States is cumulatively estimated to be 40% children and 60% adults (NIH, 2010; iData, 2010).

## Factors impacting utilization of CIs in the United States

The following seven factors contribute to low utilization of CIs by individuals who could benefit from them in the United States. Many of these same barriers are present in other countries of the world.

### Low general awareness

Awareness of cochlear implantation and its benefits in overcoming the effects of severe to profound hearing loss is low among the general population and even among health-care professionals. Low awareness follows attitudes regarding hearing loss generally, specifically the lack of understanding about the significant effect of not hearing on an individual's life. For a child, even a mild hearing loss can interfere with academic potential. For a working age adult, hearing loss can compromise employment opportunities, family relationships, and social–emotional well being. For an older adult, untreated hearing loss has been associated with higher rates of dementia (Lin, 2012). Despite this, too often hearing loss is viewed more as an annoyance than as a significant health issue.

Unlike many other health issues such as diabetes and breast cancer, there are no ongoing, major national awareness campaigns that reach out to the media and the general population about hearing loss broadly or about cochlear implantation specifically. When there is media coverage of cochlear implantation, it often focuses on the controversy surrounding its use, generated by some members of the Deaf Community. Hence the messages about the benefits of the intervention can be confusing to the general population. Many CI professionals note that when they mention to acquaintances what they do, they are often met with comments like: ‘Aren't cochlear implants controversial?’ or ‘Do they really work—I've heard there is opposition to them from many deaf people.’

There are many organizations in the field representing individual interest groups including organizations of adults with hearing loss, parents of hearing impaired children, professionals who provide services to families, clinicians representing various professions (surgeons, audiologists, speech pathologists, educators, and therapists), scientists, and manufacturers of hearing technology. Each interest group or professional category has its own membership and point of view and carries out its own individual activities and methods of outreach. Though some organized campaigns do exist, such outreach activities tend to focus on fundraising and organizational awareness rather than on an overriding public policy issue. Certainly none of these efforts by national organizations highlight cochlear implantation as a safe and effective health intervention for deafness benefiting children and adults.

### Hearing loss referral networks are unaware of candidacy and outcomes

Anecdotal information indicates that many (if not most) medical school programs do not address cochlear implantation as part of the study of hearing loss. As a consequence internists, family doctors, pediatricians, and gerontologists—all primary care physicians who have occasion to see and advise patients who are potential candidates to seek an evaluation for a CI—do not receive even basic information in medical school on the intervention. This lack of training may well impact on whether or not primary care physicians provide basic information and guidance to their patients who may be CI candidates.

Pediatricians can play a crucial role in providing information and making referrals for appropriate children with hearing loss in a timely fashion. Yet in one study, over one-third of pediatricians experienced with treating children with sensorineural hearing loss indicated that such physicians are uncomfortable in discussing CIs and counseling families about them (Mathews *et al*., 2009).

The fact that physicians are not providing such information on CIs sometimes reflects a hesitancy to encourage patients to pursue hearing solutions generally. Only 14.6% of primary care physicians conduct hearing screenings of any type (e.g. tuning fork) of their patients (Kochkin, 2009).

Even specialists in hearing impairment such as audiologists and hearing aid dispensers may be unaware of CI candidacy criteria or typical outcomes with a CI and are consequently uncomfortable about making referrals (Hogan *et al*., 2001). In a survey conducted at the annual conference of the American Academy of Audiology in 2008, 437 attendees completed a survey regarding patient referrals for cochlear implantation. Relatively few of the audiologists interviewed indicated that they had made a CI referral; 20% noted that they had not seen a candidate for cochlear implantation in their practice in the prior 6 months. Fifty-five percent noted that they had seen fewer than five CI candidates in the prior 6 months (Huart, 2009).

In another survey of 70 general ENT physicians practicing in the United States, 80% indicated that they had received training on CIs during their residency or fellowship years. The same group of ENTs, reporting on a total of 13 662 patients with moderate to profound bilateral sensorineural hearing loss, indicated that they had referred 3% of those patients for a CI evaluation (Frost and Sullivan, 2008). EI professionals provide services to families of children under three years of age who have identified disabilities. Such professionals are the common denominator for all families seeking information after a child has been identified with hearing loss under newborn hearing screening programs. EI can serve to explain, help initiate, and expedite diagnostic and treatment services for young deaf children. A number of studies have examined the extent to which such professionals are familiar with cochlear implantation and further whether or not they alert parents to their child's possible candidacy. One study surveyed EI professionals to determine their familiarity with key CI topics. When asked the youngest age at which a child with profound hearing loss could receive a CI according to the FDA criteria, 67% of EI professionals in the sample of professionals from five United States cities (*n* = 150) responded correctly by indicating 12 months of age. The same survey asked the group of EI professionals to indicate the age at which children who were CI candidates at birth should be implanted to derive maximum benefit from the CI intervention; 57% gave the correct answer of 18 months (Sorkin, 2010).

A study by Sorkin and Zwolan (2008) examined a random and statistically significant sample of parents of CI children six years of age and younger (n = 148) to assess information provided to the family by their EI professional regarding their child's possible candidacy for a CI. Thirty-one percent indicated that they had received information on CI from EI. That study also found that most parents judged the information received on ‘communication options’ to be biased in some fashion – either somewhat focused or biased on one option (49%) or very biased for one approach or option (18%). Fifty-seven percent indicated that the bias was for a sign-based approach.

The problem of not referring patients who could potentially benefit from CI is widespread and is an issue among those who are specialists in hearing loss as well as those who are not. The problem affects CI candidates of all ages and interferes with utilization rates as well as the time that it takes for appropriate candidates to learn about the intervention and move through the system to receive a CI.

### Political issues associated with deafness

The two issues above – awareness and referral behaviors – are complicated by the political complexities of deafness in the United States. The Deaf Community promotes early use of sign language for all children with hearing loss and has been hesitant to fully embrace CIs for infants as a means to provide access to sound. The Deaf Community (with a capital ‘D’) refers to individuals who use American Sign Language and view themselves as being part of a community who believe that their deafness is an important part of their self-identity. Ten years ago (and even to this day to a lesser degree) many individuals from the Deaf community encouraged parents to wait until a child reached the teen years to pursue surgery so the deaf adolescent could decide if (s)he wanted to receive a CI. Some individuals from the culturally Deaf Community continue to advocate for waiting to implant.

In 2001, the National Association of the Deaf (NAD), the organization representing deaf signing people stated the following in an online dialogue called ‘Cochlear Implants—The Debate’: ‘implants are seen as the panacea for deafness’ and ‘research outcomes are so positive that one must pay attention to the other side of the coin’ (PBS, 2001). Such comments from an active and respected national organization with global visibility served to confuse parents, professionals, and the public at large regarding the benefits of pediatric cochlear implantation.

In 2012, the Audism Free America group continues to oppose CIs and spoken language approaches for deaf children. The group's five stated demands of ‘EHDI’ (defined by this group as Early Healthy Deaf Identification – a play on the term Early Hearing Detection and Intervention) include the mandate that EHDI shift its approach to Deaf infants and their families from a pathological/medical bias to a positive/culturally additive model in identification. To ensure this, parents need to be informed at the time of identification that American Sign Language is a linguistic human right for a Deaf child’ (Audism Free America, 2011). The group has picketed a number of national meetings that provide educational sessions on hearing technology and spoken language development in children. NAD's president joined one such demonstration in 2011 and aligned with the Audism Free movement noting that all deaf children should grow up learning American Sign Language and using it in their educational environment (NAD, 2011).

The perspective that all deaf children need sign language—regardless of whether or not they use technology—is confusing to both parents and professionals and continues to impact the EI process. In 2010, representatives of the American Cochlear Implant Alliance and other groups met with the US Department of Education to urge that comprehensive and accurate information on cochlear implantation be provided to all parents of children with severe to profound hearing loss. The meetings did not result in any guidance or information being issued by the Federal government regarding the benefits of early implantation as officials feared that doing so would suggest they were supporting one communication option over another.

As a consequence, baseline information about options provided to parents of deaf children varies considerably by state and even within individual states. Such variation was confirmed by two parent surveys conducted in 2003 and 2011. Parents of young children who had received CIs were asked about the information and services that their family received under the federally funded EI program. Less than a third of parents of implanted deaf children indicated that they received specific information on their child's possible CI candidacy from their EI professional. Information that helped them make their decision regarding CI for their deaf child came from other sources—audiologists, physicians, parents, parent organizations, or the Internet. This was the case in 2003 and was still true 7 years later (Sorkin and Zwolan 2008, 2012).

Such caution (or even outright opposition in some cases) regarding CI for young children has affected EI programs throughout the United States to the extent that parents often receive no information about the outcomes and benefits associated with early implantation.

In the early 1990s, the intervention was characterized by some deaf people as suspect for adults as well as children. The author of this paper was implanted in 1992 and was frequently subjected to negative commentary such as ‘You are denying your deafness by pursuing a cochlear implant’ or ‘Why don't you learn sign language and acknowledge that you are part of the Deaf community?’ Such attitudes on cochlear implantation for people of all ages contribute to negative perspectives about the intervention among members of the public.

### Clinic and hospital financial issues

At the time this article was written, there were three main types of health insurance in the United States. Private employer-based health insurance plans are the most common with approximately 60% of Americans (US Census, 2008) covered by private or commercial plans. The majority of employers in the United States offer health insurance to an individual employee of a company and usually his or her family. In general, the employer pays for a portion of the individual plan and sometimes a percentage of the family plan though there is great variability in the amount of employer/employee cost sharing. Generally there are choices available as to the type of insurance plan the employee may enroll in. For example, some people prefer to make a greater contribution to their health plan cost and have the ability to visit any physician or provider they choose. Other individuals are willing to limit their selection of health-care providers to those who are part of a network and thereby pay a lower premium for their health insurance.

The second main category of health insurance plans are those provided by the government for eligible individuals. These include Medicare (for people age 65 and older or people younger than 65 years if they have a disability) and Medicaid (for low-income families who meet certain income criteria). Adults on Medicaid are often disabled, low-income people. Medicaid in some states covers any child with a disability regardless of parental income. Children are covered by Medicaid for unilateral cochlear implantation in all 50 states; there is variability in coverage for bilateral CIs by Medicaid. Adults are another matter; with budget-cutting pressure on Medicaid programs nation-wide, some states are removing coverage of cochlear implantation for adults as it is considered an ‘optional’ service under the federal guidelines. Advocates argue that many adults are unable to work and rely upon Medicaid benefits because of their deafness. CI clinicians report that many adults who receive CIs under Medicaid return to the workforce with their restored hearing, becoming self sufficient and no longer needing public support. Hence, advocates argue that CIs received by deaf adults on Medicaid ‘pay for themselves.’

A third category of health plans includes public plans for military or former military members and their immediate families (e.g. Tricare and Veterans Administration) and plans covering federal employees and their immediate families. All such public employer plans cover unilateral and bilateral cochlear implantation for appropriate children and adults as well as the necessary follow-up care.

In the United States, private (employer)-based insurance as well as public health programs generally cover the implant device and related surgical costs. Audiology and habilitation follow-up are also covered by most plans. Increasingly, bilateral cochlear implantation is covered by private plans. Although there is some variation, the majority of private plans do cover bilateral CIs. This is an important access improvement from the late 1990s when many private plans failed to cover unilateral CIs.

The major insurance issue in the United States is increasingly the amount reimbursed to the hospital or clinic for various elements of the CI intervention, particularly by Medicaid programs which are operated with considerable discretion by individual states. In some states, the reimbursement to the hospital for the CI device and related surgical costs from the Medicaid program covers less than 10% of the actual cost of the device (State Medicaid Websites, 2011, unpublished raw data).

In states with large numbers of Medicaid children and low payment rates, there have been instances in which hospitals have closed pediatric programs because of the large financial losses associated with operating the program. Such closures limit access to care for all children but especially for children from families with fewer financial resources who may not be able to travel to distant locations for the implant surgery and needed follow up appointments for programming and therapy (Sorkin and McClanahan, 2002; Rand Corp, 2002; Davidson, 2011).

Some hospitals may contain costs by limiting the number of poorly reimbursed surgeries. Although there are no published studies on this topic and CI programs may not publicly admit to using this approach, some clinicians have confidentially noted that they were instructed by their hospitals to limit the number of Medicaid surgeries by putting patients in a queue.

Apart from Medicaid, surgery for a CI is generally reimbursed fairly by private insurers and by Medicare. The aftercare reimbursement for programming and therapy is more variable and is often a function of how aggressively the clinic or hospital has negotiated payment rates with private insurance companies. Given the skill and training required for CI programming, for example, audiology reimbursement is often quite low. Additionally some aspects of aftercare, such as troubleshooting the sound processor, may not be covered by some insurance plans and hence the cost of a CI clinician's time must be absorbed by the clinic (Tong, 2009).

The reimbursement amount for post-operative CI therapy performed by a trained professional with a Masters level degree (such as a speech pathologist) can vary depending upon the individual payer and/or the manner in which the clinic has pursued and negotiated reimbursement for such services. Both Medicare and Medicaid typically reimburse poorly for therapy services. Hence the mix of different payers for patients at any particular CI clinic can impact on the financial picture associated with therapy reimbursement. In some instances, if therapy services are losing money for the clinic or hospital, this can discourage the hiring of additional therapeutic staff and cause long patient waits for services or fewer visits per patient. Some clinics make up financial losses by raising money through foundations or by seeking donations.

Another type of insurance challenge is an arbitrary limitation on the number of therapy sessions covered by one's health insurance provider. For example, Colorado Medicaid limits to 20 per year the number of therapy sessions that can be provided to a child post CI. For many young children, this may not be sufficient in the first year or two after surgery. Private insurance policies sometimes place similar arbitrary limits on the amount of covered therapy. Often a physician's letter of necessity can be utilized to successfully address such stated limitations though some families become discouraged and simply accept what is in the policy without trying to overturn a restrictive approach.

### Need for widely accepted ‘best clinical practices’

Standard clinical practice guidelines have been accepted for many medical conditions, and are typically recommended widely regardless of an individual's income, education, ethnicity, or family situation. For example, there are published guidelines that are broadly understood, accepted and implemented by primary care physicians regarding screening for colorectal cancer for individuals with high risk or normal risk. Because of this, follow-up after a colonoscopy is guided by accepted practices that are widely known. The same is true for screening, follow-up and management for breast cancer, diabetes, heart disease, high cholesterol, and a host of other medical circumstances. We are conditioned by our physicians, our friends and family, and by the media to adhere to specific practices.

The audiology community has published advice about CIs and their place in an audiology practice. The American Academy of Audiology (2000) published its ‘Audiology Clinical Practice Algorithms and Statements’ in the *Audiology Today Special Issue*. The statement includes a section on CI assessment with Common Procedural Terminology codes and a flow chart of the typical assessment procedure used for adults. It does not, however, provide specific guidance that could be used by medical professionals in and out of the hearing loss field to guide candidate referral.

*Preferred Practice Patterns for the Profession of Audiology* was published by the American Speech-Language-Hearing Association (ASHA) (2006). It includes preferred practice patterns for cochlear implantation but lacks specificity that can be readily applied by clinicians. For example, the ASHA resource mentions the importance of ‘regularly scheduled follow-up visits to ensure appropriateness of speech processor map(s) and integrity of the cochlear implant’ though there is no definition of what the follow-up schedule should be. Both the Academy and ASHA resources provide comprehensive discussion but not enough specificity to be considered as best clinical practices.

A 2010 text (Wolfe and Schafer, 2010) offers practical guidance for clinicians who program CIs, introducing the basics of CI programming and continuing through advanced programming techniques. While it provides one of the first comprehensive guides on programming, use of assistive devices and management of the CI patient, this work cannot be considered a clinical guideline.

Both the Academy and ASHA resources provide comprehensive *general* discussion by professional societies in the field of audiology. In 2012, there were no published or accepted guidelines for best clinical practices for either children or adults.

For children, one would expect that best clinical practice guidelines would include:

- age of implantation to achieve best outcomes;
- habilitation practices including quantity and type;
- school support;
- appropriateness of cochlear implantation in the child with other medical conditions;
- appropriateness of cochlear implantation for children from diverse socio-economic situations; such as English as a second language, low parent education or income level.

For adults, one would expect best clinical practice guidelines to address:

- effect of duration of deafness and amount of residual hearing as key factors in outcomes;
- rehabilitation practices, both the need for rehabilitation and the nature of such follow-up for adults of all ages;
- best practices for treatment of older adults including special training on the sound processor

The lack of such specific clinical guidelines exacerbates efforts to have cochlear implantation be widely recognized as the standard of care for deafness.

### Timely and comprehensive cost-effectiveness data

There is an extensive body of research demonstrating the cost-effectiveness of CIs in children and adults. The majority of such studies were completed at least 10 years ago such as the seminal research by Cheng *et al*. (2000) demonstrating cost utility in children and work by Francis *et al*. (2002) highlighting benefits for older adults. A study by Wyatt *et al*. (1996) compared the cost of cochlear implantation to other common medical interventions such as tuberculosis testing and hypertension treatment by assessing cost per quality-adjusted life year (QALY). CIs compared favorably with these common medical procedures.

More recent studies of societal benefit are needed, especially research demonstrating the broader payback of the intervention. It is well known, for example, that hearing loss has a negative impact on earnings and employment. Hearing aids have been found to mitigate the effect for those with moderate to severe hearing loss by 65–77% (Kochkin, 2010). Kochkin's study also found that those with severe hearing loss who did not use hearing aids had unemployment rates that were nearly double that of those who did use amplification (15.6 versus 8.3%). We have no such data for individuals who use CIs though the information would be valuable in making a case for coverage of the intervention under state Medicaid programs. At present, adult Medicaid coverage in the United States is quite variable.

The public education system in the United States has not evolved to meet the needs of this new population of deaf children, many of whom have CIs. Rather, many states continue to operate state residential schools for the deaf where costs have risen to nearly $100 000 per year, per child (State education reports). Many families are unable to secure appropriate educational options for their children with CIs – children who listen and speak but are deaf and still need specific services. Studies comparing outcomes of these two groups of children and the long term benefits of CIs are increasingly available but have not yet become part of the public decision-making for our educational system.

To help make the CI intervention the standard of care, we need to complete additional studies and make the results of such studies available to policy-makers who affect decisions on insurance coverage, educational opportunities, and advisement practices for EI.

### Need for a dedicated organization focused on CIs

Unlike many other health issues such as diabetes and breast cancer, there are no ongoing national awareness campaigns that reach out broadly to the media and the general population about hearing loss. There are many organizations in the field representing individual interest groups including adults, children, clinicians, scientists, and manufacturers of hearing technology. Each has its own perspective and special interest. There is no one comprehensive organization that seeks to bring together the various parties and interests, increase public knowledge and promote positive appropriate public policy about hearing loss. This issue may soon be remedied by the creation of a new organization in the field, the American Cochlear Implant Alliance (ACI Alliance). The following article by founding co-chairs John Niparko and Teresa Zwolan and strategic consultant Robin Harding provides background on this new organization.

## Conclusions

Numerous studies have demonstrated that cochlear implantation provides cost effective results in children and adults. Recipients and parents often characterize the outcomes afforded by CIs to be life-changing, even miraculous. Ironically, the intervention is remarkably underutilized in the United States with only 5% of eligible candidates (adults and children) receiving a CI. Utilization rates have not changed much over time except in the pediatric age group. When examined in a standard-of-care context (e.g. Is CI routinely considered and does the medical community refer appropriate individuals for this procedure?), CI utilization patterns indicate that the intervention has not yet become an accepted best practice for deafness.

Seven factors have been identified as contributing to the low utilization by individuals who could benefit from a CI in the United States. Many of these same barriers are present in developed and developing countries around the world. Awareness about the benefits of CIs for both adults and children remains low despite the fact that this technology first received FDA approval nearly 30 years ago. The absence of major awareness campaigns about hearing loss broadly and cochlear implantation specifically contributes to this low public awareness.

For most medical interventions, primary care physicians are a critical link in the referral process connecting patients to appropriate medical specialists. Yet studies of referral patterns have demonstrated that most clinicians are unsure of whether and when to refer a patient with hearing loss to a CI clinic. Anecdotal information indicates that CIs are often not addressed as part of the medical school curriculum on hearing loss.

Both of these factors – public understanding and inconsistent referrals by clinicians – are complicated by the long-standing political issues associated with deafness. A number of groups representing Deaf culture continue to oppose the use of spoken language approaches for deaf children and press for sign language instruction for all children with hearing loss. Such perspectives add controversy to the referral process and serve to confuse a wide range of individuals about typical outcomes associated with cochlear implantation. A 2012 article published in *Pediatrics in Review*, the online publication of the American Academy of Pediatrics, claimed to address the ‘ethics of cochlear implants’ for pediatricians (Lantos, 2012). There was no mention of recent studies showing the benefits provided by cochlear implantation for a deaf child in terms of speech and language development, literacy, and life opportunities. Rather, the article raised questions about the cost and extensive post-operative care and variability in outcomes, reiterated debates from 100 years ago about whether deaf parents should be allowed to have children, and reminded the reader of the controversy associated with use of genetic testing. Such an article in a publication of a prestigious clinical society is illustrative of the type of information that causes public confusion and inconsistent referral patterns by physicians.

In 2012, the primary financial concern for clinics and hospitals related to cochlear implantation includes reimbursement levels for services such as programming and therapy and certain functions like troubleshooting the sound processor. (The latter is typically not covered by health insurance.) In addition, Medicaid is quite variable at the state level with some states not paying for processor replacements, bilateral CIs, or sufficient amounts of therapy services for children.

Standard clinical guidelines have been accepted and published for many other health issues to guide primary care physicians and others in the screening and follow up care of patients. This has not been the case for cochlear implantation, which further contributes to the referral patterns discussed in this paper.

Cost-effectiveness studies have demonstrated that cochlear implantation is highly cost-effective in both children and adults. However, such studies were completed 10 or more years ago. Recent studies that document the broad life benefits of CI such as literacy and language of deaf children and the relative cost of deaf education versus mainstreaming with appropriate support are urgently needed. For adults, there have been no comprehensive studies on the impact of CI on the employment and advancement of working age adults. There are also no studies documenting the general benefits to society of providing hearing to those who have lost it or who were born deaf. Having such data would provide additional impetus for timely referrals for people of all ages.

Finally, all of these factors could have been more effectively advanced were there an organization focused on cochlear implantation. With the incorporation of the American Cochlear Implant Alliance (ACI Alliance) in 2012, there is now such a body to press for all of the above changes in an organized manner. Such an organization is needed to help make the CI intervention available to the many children and adults who could benefit in the United States.
